# Increased expression of the *MALE STERILITY1* transcription factor gene results in temperature-sensitive male sterility in barley

**DOI:** 10.1093/jxb/eraa382

**Published:** 2020-08-21

**Authors:** José Fernández-Gómez, Behzad Talle, Zoe A Wilson

**Affiliations:** 1 School of Biosciences, University of Nottingham, Sutton Bonington Campus, Loughborough, Leicestershire, UK; 2 University of Toronto, Canada

**Keywords:** Anther, barley, hybrid, male sterility, MS1, plant reproduction, pollen, temperature

## Abstract

Understanding the control of fertility is critical for crop yield and breeding; this is particularly important for hybrid breeding to capitalize upon the resultant hybrid vigour. Different hybrid breeding systems have been adopted; however, these are challenging and crop specific. Mutants with environmentally reversible fertility offer valuable opportunities for hybrid breeding. The barley *HvMS1* gene encodes a PHD-finger transcription factor that is expressed in the anther tapetum, which is essential for pollen development and causes complete male sterility when overexpressed in barley. This male sterility is due at least in part to indehiscent anthers resulting from incomplete tapetum degeneration, failure of anther opening, and sticky pollen under normal growth conditions (15 °C). However, dehiscence and fertility are restored when plants are grown at temperatures >20 °C, or when transferred to >20 °C during flowering prior to pollen mitosis I, with transfer at later stages unable to rescue fertility *in vivo*. As far as we are aware, this is the first report of thermosensitive male sterility in barley. This offers opportunities to understand the impact of temperature on pollen development and potential applications for environmentally switchable hybrid breeding systems; it also provides a ‘female’ male-sterile breeding tool that does not need emasculation to facilitate backcrossing.

## Teaser

Overexpression of *HvMS1* in barley results in thermosensitive male sterility, due to persistent tapetum, failure of anther dehiscence, and sticky pollen; however, fertility is rescued by growth at higher temperatures.

## Introduction

Population growth alongside increased desire for consumption has made food security an important global issue. The FAO reported that between 2011 and 2013, an estimated 842 million people (12% of the global population) were unable to meet their dietary energy requirements (FAO, 2013). This situation is likely to worsen over time as the population is expected to increase from 6 to 9 billion. This is predicted to increase food needs by 50% by 2030, and 100% by 2050 (FAO, 2013). In addition, the factors that generated major improvements in global food production during the green revolution, such as expansion of irrigation or widespread use of fertilizers, are now limited and environmentally unsustainable (Whitford *et al.*, 2013). Therefore, future yield increases need to come from more efficient and accurate breeding and selection technologies. Among these, hybrid seed production is one of the most attractive since it exploits heterosis (hybrid vigour) to give increased yield and resilience, without the requirement for additional resource inputs (Shull, 1908). The mechanism that causes heterosis is still unclear; however, this enhanced germplasm is generated as a consequence of outcrossing as opposed to inbreeding (Whitford *et al.*, 2013). Estimates of heterosis yield increases vary between 3% and 20% depending on the crop; this is partly due to increased production but also to enhanced biotic and abiotic resilience (Longin *et al.*, 2012). However, to exploit heterosis in autogamous plants, such as wheat and barley, self-fertilization needs to be controlled via the generation of male-sterile plants for crossing, and these lines need to be subsequently maintained (Omarov, 1976; Virmani, 1994). Additional features also need to be considered for a successful hybrid system, including pollen viability, flower opening/pollination timing, and the amount of pollen released (Yan *et al.*, 2009). Increasing fundamental understanding of pollen and anther development is critical for the effective application of such breeding systems to prevent failure of hybrid generation and subsequent crop fertility, particularly under variable environmental conditions.

Different approaches such as cytoplasmic male sterility (CMS) and chemical gametocides have been used to produce hybrids commercially; however, these remain a major challenge for some crops, particularly temperate cereals. Hybrid rice was first commercialized in 1976 based on a CMS system (Virmani, 1994; Yuan and Mao, 1994; Longin *et al.*, 2012). Currently, >20 Mha of rice is cultivated worldwide, 17 Mha of which in China, of which 80–90% is produced using the CMS system and the rest through temperature- or photoperiod-sensitive male sterility (Si *et al.*, 2011; Longin *et al.*, 2012). Deployment of hybrid systems in the temperate cereals has been significantly slower, with ~200 000 ha of hybrid wheat currently planted in Europe, mainly in France (160 000 ha) (Saaten-Union; https://www.saaten-union.fr/) and Germany (25 000 ha) (Saaten-Union; https://www.saaten-union.de/). Most of these hybrids are produced using chemical hybridization agents (CHAs), mainly Croisor (Sintofen; formerly Dupont-Hybrinova, Saaten-Union Recherche, France) (Longin *et al.*, 2012). Of the 30 000–35 000 ha of hybrid wheat cultivated in China and India, 50% is based on photoperiodic sensitivity and the rest on a CMS system from *Triticum timopheevii* (Singh, 2010). Despite a long-term interest in hybrid barley (Suneson, 1940), it was only recently that hybrid barley ‘Colossus’ based on the CMS system (Ahokas, 1979; Longin *et al.*, 2012) was commercialized by Syngenta; currently >200 000 ha of this is cultivated in the UK, France, and Germany (Longin *et al.*, 2012).

Hybrid breeding systems that utilize the chemical gametocide Croisor target the development of the anther tapetum, which is critical for functional pollen development. The tapetum regulatory network involves a number of key transcription factors (DYT1, TDF1, AMS, MS188, and MS1; Zhu *et al.*, 2011), which have been characterized in Arabidopsis, but are also conserved in cereals, such as rice (Wilson and Zhang, 2009) and barley (Fernández Gómez and Wilson, 2012). For example, two transcription factor genes, *OsUTD1* (*UNDEVELOPED TAPETUM*) and *OsTDR*, have been identified as the rice orthologues of the Arabidopsis *DYSFUNCTIONAL TAPETUM1* (*DYT1*; Zhang *et al.*, 2006) and *ABORTED MICROSPORE1* (*AMS*; Sorensen *et al.*, 2003) genes, respectively. *OsUTD1* encodes a basic helix–loop–helix (bHLH) protein, which acts after tapetum initiation in an analogous manner to *AtDYT1* (Jung *et al.*, 2005), whilst *OsTDR* (Li *et al.*, 2006; Zhang *et al.*, 2008) has been shown to play an important role during rice tapetum development, lipid transport, and metabolism for pollen wall formation.

Previously we identified the *HvMALE STERILITY1* (*HvMS1*) PHD-finger transcription factor gene that is transiently expressed in the tapetum prior to the pollen mitosis I (PMI) stage (Fernández Gómez and Wilson, 2014), which is essential for pollen development in barley and orthologous to Arabidopsis *MS1* (Wilson *et al.*, 2001) and rice *PTC1* (Li *et al.*, 2011). The Arabidopsis *ms1* mutant is completely male sterile; however, RNAi silencing of *HvMS1* in barley only showed partial sterility, whilst overexpression of *HvMS1* caused complete sterility without impacting on other aspects of plant development (Fernández Gómez and Wilson, 2014).

Here we describe characterization of *HvMS1*-induced sterility and report an interesting temperature sensitivity of this phenotype, which we believe is the first example of environmentally restorable male sterility in barley. This temperature sensitivity offers mechanisms to help understand the impact of high temperature during pollen development. It also has potential application for hybrid generation by combining the complete sterility of *HvMs1* overexpression lines and their thermosensitivity, enabling effective fertility control and subsequent recovery. Lines carrying a single copy of the *HvMS1* overexpression transgene, which will segregate 1:1 when outcrossed, also provide a tool for traditional breeding and research. By using this male-sterile line as a female parent in breeding programmes it can simplify the cross-pollination process, avoiding the need for emasculation and reducing time and effort in back-crossing, and eliminating selfing contamination.

## Materials and methods

### Plant materials

Barley (variety Golden Promise) *HvMS1* overexpression (*HvMS1OEx*) and *HvMS1RNAi* lines were generated previously (Fernández Gómez and Wilson, 2014). Five independent overexpression lines were produced (Lines A1, B1, C1, D1, and E1); all showed the same phenotypes, and Lines A1 and B1 were selected for further detailed analysis. T_0_ overexpression lines were maintained by crossing with Golden Promise wild type (WT) to produce F_1_s; these were maintained by selfing at >20 °C to produce F_2_ generations (Supplementary Fig. S1 at *JXB* online shows the constructs used). F_1_ generations were sown alongside WT and T_2_*HvMS1RNAi* lines, and grown under controlled conditions [15 °C/12 °C; 16 h photoperiod; 80% (v/v) relative humidity, 500 µmol m^–2^ s^–1^ metal halide lamps (HQI) supplemented with tungsten bulbs]. Plants were grown in 5 litre pots containing Levington C2 compost (three plants each); the F_1_ generation of single-copy male-sterile mutants were crossed with WT or T_2_*HvMS1RNAi* plants to generate seed for analysis.

Three barley varieties, RAGT Planet, Moonshine, and Optic, were grown (15 °C) alongside Golden Promise *HvMS1OEx* lines for crossing. F_1_s from the first cross were grown for three more generations and crossed into the corresponding background to introgress the transgene into the elite backgrounds. Fertility recovery of positive overexpression lines was conducted at 20 °C.

### Genotyping analysis

Genomic DNA was extracted using the ISOLATE II Genomic DNA Kit (Bioline) from WT and transgenic plants. Transgenic plants, *HvMS1OEx*/*HvMS1RNAi* lines, were genotyped using primers pBract214F and HvMS1-3R (Supplementary Table S1) for the overexpression and the RNAi sense fragment (Supplementary Fig. S1). Crosses between *HvMS1OEx* and *HvMS1RNAi* lines generated two amplification products when both transgenes were present (OEx and RNAi sense, 971 bp and 652 bp, respectively; Supplementary Fig. S1), or single products when only one was present. The antisense target sequence was also analysed by PCR [Primers Iv2F and NostermR (Supplementary Table S1), 719 bp product; Supplementary Fig. S1].

### Quantitative real-time PCR (qRT-PCR) and copy number determination

cDNA was synthesized according to Fernández Gómez and Wilson (2014). Gene-specific primers, HvMS1-1F and HvMS1-3R (Supplementary Table S1), were used for qRT-PCR according to Yang *et al.* (2007), using Brilliant SYBR Green qPCR master mix (Stratagene) and the LightCycler®480 Real-Time System (Roche). Expression was normalized using the α-tubulin gene (Supplementary Table S1) (50 cycles; 63 °C annealing temperature). Transgene copy number was calculated by qRT-PCR (Shepherd *et al.*, 2009). Genomic DNA, extracted from leaves, was diluted to equal concentrations and the hygromycin gene was amplified (HygF/R; Supplementary Table S1) (50 cycles; 64 °C annealing temperature). Four known samples were used as controls (Hyg copy number determined by Genetics Ltd, JIC Norwich).

### Fertility analysis

Plant fertility was calculated using 6–10 spikes per plant. The number of total florets per spike was counted as well as the seeds present in the spikes. Fertility was calculated as a percentage by counting the number of seeds compared with the total number of florets per spike. Pollen viability was determined using Alexander stain according to Fernández Gómez and Wilson (2014).

### Temperature sensitivity analysis

Temperature sensitivity experiments were conducted in controlled-environment (CE) rooms at 15/12 °C and 20/15 °C, 16 h photoperiod, and in a glasshouse at temperatures >25°C during the period March–September, using overexpression lines A1 and B1 (F_2_ generation). Temperature was recorded using a data logger.

To study the stage sensitivity to temperature changes, *HvMS1OEx* B1 line plants (F_2_) were grown at 15/12 °C with a 16 h photoperiod. Individual tillers were staged (Zadoks *et al.*, 1974; Fernández Gómez and Wilson, 2012) and transferred to a CE room at 20/15 °C and 16 h photoperiod, or to a glasshouse at temperature ≥25 °C. Plants transferred before main tiller stage 30 were staged only using the main tiller, and fertility was counted as a whole plant. From stage 30, main tillers as well as individual tillers in the plants were staged, and fertility was calculated for each tiller independently.

### Microscopy

Staged florets from the central zone of the spike were collected and dissected for light microscopy (Fernández Gómez and Wilson, 2012). Florets were fixed and embedded in Technovit or Spurr resin. Floral panicles were fixed overnight in 4% (v/v) paraformaldehyde (rotating, 4 °C). Samples were brought to room temperature and tissues were washed twice (30 min each) with 1× phosphate-buffered saline. For Technovit, fixed panicles were immediately dehydrated with ethanol using a mixture of ethanol/resin (Technovit 7100; Heraeus Kulzer, Wehrheim, Germany) at increasing proportions of resin hardener I (2:1, 1:1, and 1:2) for 1 h, finishing with 100% resin. Hardener I was mixed with hardener II and samples were embedded as specified by the supplier. Samples were then hardened and mounted on a plastic block ready for sectioning. Sections were stained with 0.05% (w/v) toluidine blue prior to imaging. For Spurr resin, after 100% ethanol, samples were transferred to ethanol/propylene oxide 1:1 and then 100% propylene oxide, followed by Spurr pre-infiltration, propylene oxide/Spurr, 1:1, and finally 100% Spurr resin prior to capsule preparation.

## Results

### Male fertility of the *HvMS1* overexpression line is restored by an *MS1-RNAi* silencing transgene

Heterozygous F_1_ barley lines carrying a single copy of the *Ubi*:*HvMS1* overexpression transgene (*HvMS1OEx*) were previously found to be completely male sterile under standard growth conditions (15/12 °C, 16 h photoperiod) (Fernández Gómez and Wilson, 2014). Further analysis of these lines was conducted to understand the nature of the sterility and fertility rescue. *HvMS1OEx* heterozygous plants were slightly smaller, but appeared generally equivalent to the WT, except they were male sterile (Supplementary Fig. S2). However, homozygous lines carrying two copies of the *HvMS1OEx* transgene showed more severe phenotypes, producing multiple tillers which were completely male sterile, with extremely stunted vegetative growth (~50% WT height; Supplementary Fig. S2) and spikes that did not extrude out of the flag leaf. Heterozygous F_1_ barley lines carrying a single *HvMS1OEx* transgene were crossed with either the WT or a *HvMS1-RNAi* silencing line (Fernández Gómez and Wilson, 2014) to reduce *HvMS1* overexpression and determine the role of *HvMS1OEx* in the observed male sterility. Genotypes were confirmed by PCR for presence of the *HvMS1OEx* and the RNAi silencing transgenes; sense and antisense fragments were amplified separately to confirm the structure of the RNAi constructs since a number of lines were found to be deleted for one insert arm (Supplementary Fig. S1).

**Fig. 1.**
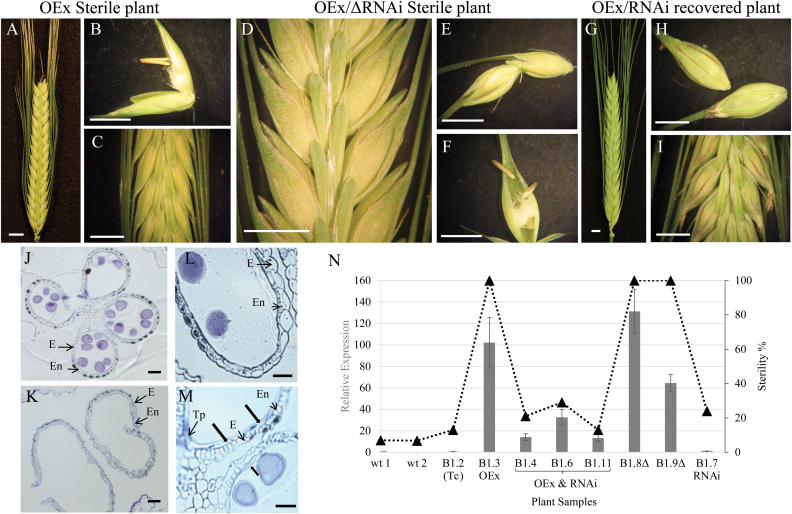
Fertility restoration of *HvMS1* overexpression lines by the *HvMS1RNAi* transgene. (A–C) Sterile barley spike from the *HvMS1OEx* line [Line B1.3 (WT×heterozygous *HvMS1OEx*)]. (D–F) Sterile barley spike containing the *HvMS1OEx* transgene and a deleted *HvMS1RNAi* construct (Line B1.8; Δ: deleted RNAi transgene); the ΔRNAi construct was non-functional and the plant was sterile due to *MS1* overexpression. (G–I) Fertility rescue in the *HvMS1OEx* line due to down-regulation of *MS1OEx* by a functional *HvMS1RNAi* construct (Line B1.6). (J) Anther section from Line B1.8 containing *HvMS1OEx* and a deleted *HvMS1RNAi* transgene. The line had indehiscent anthers and was completely male sterile. (K) Anther section from Line B1.4 containing *HvMS1OEx* and the functional *HvMS1RNAi*; fertility was rescued with normal anther dehiscence. (L) WT anther section at dehiscence; only the epidermis and endothecium layers were present. (M) Line B1.8 (*HvMS1OEx* with deleted RNAi construct) was sterile with indehiscent anthers. At anthesis, partial remains of the tapetum were still visible (thick black arrows). E, epidermis, En, endothecium, Tp, tapetum. Scale bars: (A–D) 0.1 mm; (E, F): 0.05 mm. (N) qRT-PCR and sterility analysis in control and heterozygous *HvMS1OEx* lines. The WT and non-transformed controls (Tc; Line B1.2) showed native, low levels of *HvMS1* expression and were fully fertile, whereas OEx lines (Line B1.3) showed high levels of *HvMS1* expression and associated high sterility. *HvMS1* expression was reduced in the *HvMS1OEx* lines carrying a functional RNAi construct (B1.4, B1.6, and B1.11), and these lines showed recovery of fertility. Complete male sterility was observed in the *HvMS1OEx* lines carrying a deleted, non-functional RNAi construct (Lines B1.8Δ and B1.9Δ). Fertility was seen in lines carrying only the RNAi construct without the OEx construct (Line B1.7). Full genotyping information is given in Supplementary Fig. S1.

Lines carrying the *HvMS1OEx* construct showed high levels of *HvMS1* expression and were male sterile (Fig. 1; Line B1.3), whilst the corresponding WT plants and transgenic control (Tc; Line B1.2) plants, which had been through the transformation process but did not contain transgenes, were fully fertile (Fig. 1; Supplementary Fig. S1). Crosses between the heterozygous *HvMS1OEx* lines and functional *HvMS1-RNAi* lines showed reduced *MS1* expression and associated rescue of fertility (Fig. 1 (Lines B1.4, B1.6, and B1.11), whilst those carrying the deleted RNAi constructs maintained high levels of *HvMS1* expression and were fully male sterile (Fig. 1, Lines B1.8, B1.9; Supplementary Fig. S1). Early anther and pollen development in the *HvMS1OEx* sterile lines was equivalent to that of the WT until late pollen mitosis (Supplementary Fig. S3). However, late anther development was abnormal, with delayed tapetum degeneration and increased deposition of materials into the anther and pollen walls (Fig. 1J–M). The anther stomium in these lines failed to split and the locule remained closed with no pollen release (Fig. 1J, M), whilst the equivalent WT anthers were open and pollen dehisced (Fig. 1K, L). Manually opened anthers from the overexpression lines contained sticky pollen that was difficult to extract from the anthers, but was viable, based upon manual self-fertilization; clumping of the pollen meant it was not possible to determine whether the quantity of pollen was altered. Plants carrying both functional transgenes (*HvMS1OEx* and *HvRNAi*) showed reduced *HvMS1* expression and fertility restoration (Fig. 1). Plants with only the functional *HvMS1-RNAi* transgene, or the deleted *HvMS1-RNAi* transgene without the *HvMS1OEx* transgene, were fertile (Fig. 1).

### The *HvMS1OEx* sterile phenotype is temperature sensitive

**Fig. 2.**
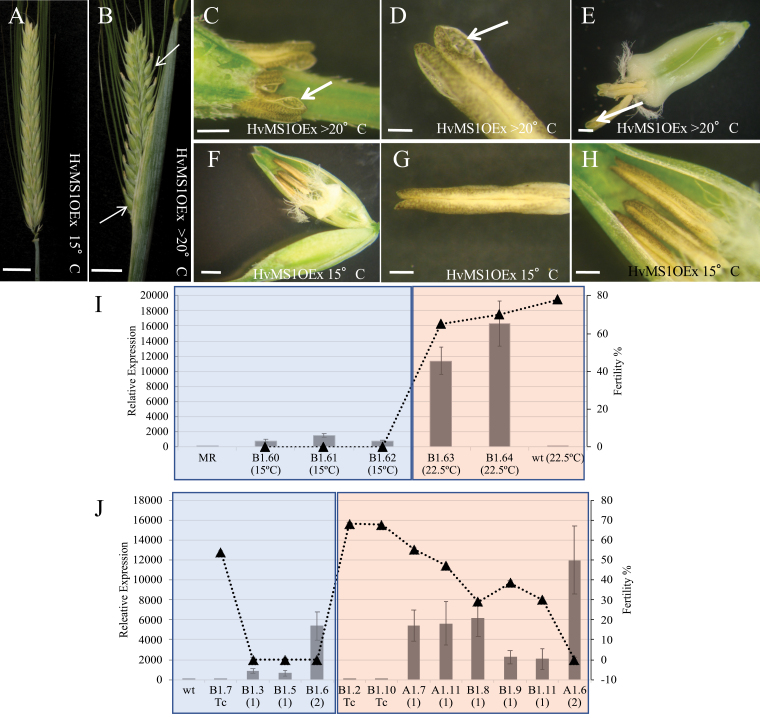
*HvMS1* overexpression lines exhibit temperature-sensitive rescue of sterility. (A) Sterile *HvMS1OEx* spike grown at 15/12 °C until flowering was completed (Zadoks stage 70). (B) Fertile spike from *HvMS1OEx* when grown at >20 °C. (C–E) *HvMS1OEx* lines show normal anther dehiscence when grown at >20 °C. (F–H) The *HvMS1OEx* line grown at 15 °C was sterile with indehiscent anthers. (I) *HvMS1* expression and fertility were determined under both temperature regimes. All lines carrying the *HvMS1OEx* transgene showed increased expression compared with the WT (MR; microspore release stage); however, lines grown at 15 °C generally showed lower expression than those at 22.5 °C and were sterile, while those grown at 22.5 °C were fertile. Expression levels are relative to native *HvMS1* expression in flowers at the MR stage in the WT. (J) *HvMS1* expression of *HvMS1OEx* lines grown at 15 °C or 22.5 °C (transgene copy number is given in parentheses). Lines grown at 15 °C (B1.3 and B1.5 heterozygous *HvMS1OEx*) showed lower expression than similar heterozygous *HvMS1OEx* lines grown at 22.5 °C (A1.7, A1.11, and B1.8). Single-copy lines grown at 22.5 °C showed similar expression to line B1.6, which has two copies and was grown at 15 °C. Homozygous *HvMS1OEx* lines (A1.6) grown at 22.5 °C showed the highest *MS1* expression. All overexpressing lines at 15 °C (B1.3, B1.5, and B1.6) and homozygous *HvMS1OEx* growing at 22.5 °C were fully sterile. Expression levels were relative to native WT *HvMS1* expression in flowers at the microspore release stage (WT). Blue shaded region, plants grown at 15 °C; red region, plants grown at 22.5 °C. Scale bars: (A, B) 1 cm; (C) 0.03 cm; (D) 0.02 cm; (E) 0.07 cm; (F) 0.1 mm; (G) 0.1 cm; (H) 0.03 cm.

**Fig. 3.**
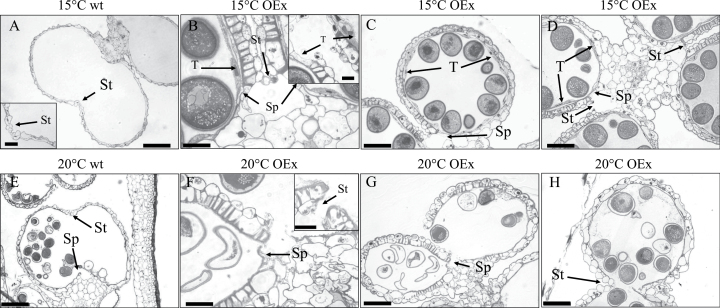
Transverse sections of anthers from WT and *HvMS1OEx* lines grown at different temperatures. (A and E) WT anthers at the anthesis stage, grown at (A) 15 °C and (E) 20 °C. (B–D) Anther sections at the dehiscence stage from heterozygous *HvMS1OEx* plants grown at 15 °C; anthers remain indehiscence and the tapetum (T) did not completely degenerate (arrows). (F–H) Anther sections at the dehiscence stage from heterozygous *HvMS1OEx* plants grown at 20 °C. Anthers dehisced normally and the tapetum (T) was completely degenerated. The septum (Sp) and stomium (St) break normally, facilitating anthesis. Scale bars: (A, B) 100 µm; (C, D, F, G) 50 µm; (E, H) 20 µm; scale bars on inset images: 50 µm.

**Fig. 4.**
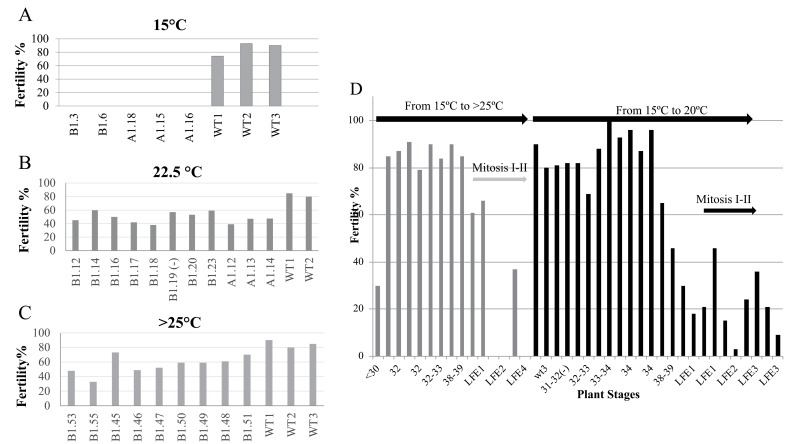
Fertility restoration of *HvMS1OEx* lines by growth at temperatures of >20 °C depends on the anther stage. (A) *HvMS1OEx* Lines (B1.3, B1.6, A1.18, A1.15, and A1.16) were fully male sterile when grown at 15 °C. The WT showed fertility of 79–95%. (B) Fertility of *HvMS1OEx* lines was restored up to 60% when grown at 22.5 °C. The negative control line for the transgene (Tc; Line B1.19) showed similar fertility to transgenic lines (59%), whilst WT lines showed up to 82% fertility. (C) Fertility restoration also occurred under a variable increased temperature regime in the glasshouse (>25 °C). (D) Individual tillers from plants growing at 15 °C were staged and then plants were moved to a glasshouse (>25 °C, grey bars) or to a controlled-environment room (20 °C, dark bars). Restoration of fertility in *HvMS1OEx* lines was higher when tillers were at stages prior to PMI/LFE1 (last flag elongation 1) for both environments, with fertility reaching close to 100%. However, after LFE2 (immediately prior to PMII), fertility restoration was lower and, although some tillers reached fertility up to 30%, in general fertility was poor or non-existent.

*HvMS1* overexpression sterile lines, containing a single copy of the transgene, were grown to grain filling (Zadocks stage 70) in CE rooms at 15/12 °C and 16 h photoperiod to determine levels of grain set. No sign of dehiscence or grain filling was observed in any of the *HvMS1OEx* transgenic plants under standard growth conditions (Figs 2A, F–H, 3B–D). However, when grown in the glasshouse or CE rooms at temperatures ≥20 °C, these plants showed normal dehiscence and seed set (Figs 2B–E, 3F–H). No significant fertility differences or anther morphology changes were seen in the WT under the different temperature regimes (Figs 2–4). Anther sections revealed that in WT lines grown at 15 °C or ≥20 °C, the tapetum degenerated completely prior to dehiscence (Fig. 3A, E); however, in the *HvMS1OEx* lines grown at 15 °C, the tapetum failed to fully break down and was still visible at the dehiscence stage (Fig. 3B–D). In addition, the anther septum and stomium did not degenerate completely in *HvMS1OEx* lines grown at 15 °C (Fig. 3B–D; Supplementary Figs S3, 4a, b). On the other hand, when *HvMS1OEx* lines were grown at ≥20 °C, the tapetum degenerated completely prior to dehiscence, the septum and stomium also degenerated normally (Supplementary Fig. S4), and restoration of dehiscence and associated fertility was seen (Fig. 3F–H). Nevertheless this rescue was not observed when transgenic *HvMS1OEx* lines had very high levels of *HvMS1* expression due to two or more copies of the *HvMS1OEx* construct; these lines were severely dwarfed, with stunted growth and abnormal anthers (Supplementary Fig. S2).

The *HvMS1* expression level was determined by qRT-PCR in a number of lines with varying *HvMS1OEx* transgene copy numbers under the different temperature conditions (Fig. 2I, J). *MS1* expression varied between the lines; those with the *HvMS1OEx* construct had higher expression than the WT or the Tc, and lines carrying two copies of the construct showed significantly higher expression (Fig. 2J). Lines with high levels of *MS1* expression were sterile at 15 °C; however, when single-copy *HvMS1OEx* lines were grown at 22.5 °C they showed fertility restoration up to ~80%. Lines with two or more *HvMS1OEx* copies with extremely high *HvMS1* expression and pleotropic phenotypes of dwarfing and abnormal growth could not be rescued at the higher temperature. *MS1* expression (native and ectopic) in the OEx lines was generally slightly increased at the higher temperatures, although line to line variation was observed (Fig. 2I, J).

Line B1.6, which contained two transgene copies (Fig. 2J), was sterile at 15 °C despite the transgene overexpression level being similar to that of the recovered single-copy lines grown at 22 °C (Fig. 2J, Lines A1.7, A1.11, or B1.8). However, all lines carrying two copies of *HvMS1OEx* showed severe phenotypes and stunting, which may impact on their ability to respond to rescue at the high temperatures. This suggests that higher temperature fertility rescue is not a consequence of the specific level of *MS1* expression, but rather a change in the development of the anther and pollen in the *MS1*-overexpressing lines at the higher temperature that compensates for the increased activity of *MS1* and thus facilitates male fertility.

To ascertain the temperature requirements for fertility restoration, three different environments were tested (16 h photoperiod): (i) CE room at 15/12 °C; (ii) CE room at 22.5/15 °C; and (iii) glasshouse with variable temperatures >25 °C. *HvMS1OEx* transgenic plants grown at 15/12 °C day/night were completely sterile (Fig. 4A); however, equivalent overexpressing plants grown at 22.5/15 °C were fertile (Fig. 4B). Their fertility restoration was at a similar level to that shown by WT or negative Tc plants (Fig. 4B). Fertility restoration of *HvMS1* overexpression lines at temperatures >25 °C was also observed (Fig. 4C).

**Fig. 5.**
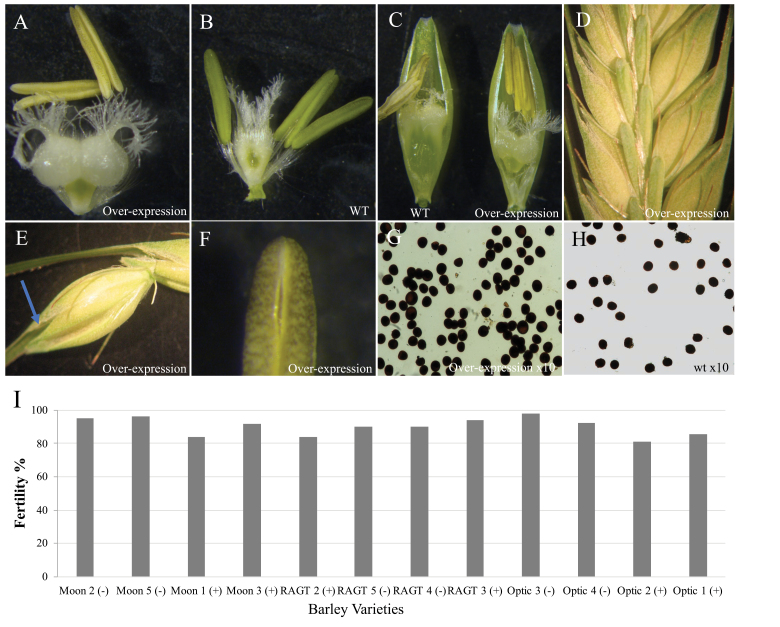
Barley *HvMS1OEx* elite cultivars showed high fertility rescue. (A, E, F) *HvMS1OEx* and (B) WT ovaries from plants grown at 15 °C. The ovaries are swollen in *HvMS1OEx* lines compared with the WT. (D, E) Floral gaping was observed in *HvMS1OEx* florets growing at 15 °C due to ovary swelling. (F) *HvMS1OEx* indehiscent anther at 15 °C partially opened under dissection microscope lights. (G, H) *HvMS1OEx* lines contained viable pollen (G) equivalent to WT (H). (I) Elite barley lines, Moonshine (Moon), RAGT Planet (RAGT), and Optic, containing the sterile *HvMS1OEx* lines showed fertility recovery of >80% when grown at >22 °C. Positive for the overexpression transgene: (+); negative for the overexpression transgene (–).

Closer analysis of *HvMS1*-overexpressing sterile florets revealed ovary swelling compared with the WT (Fig. 5); this swelling pushed the lemma and palea apart, opening up a gap that exposed the ovary to cross-pollination. Attempts to pollinate overexpression sterile lines using manually excised sterile line anthers were complicated by indehiscence and pollen stickiness making pollination extremely difficult. Nevertheless, sterile anthers collected from overexpression lines at 15 °C when kept within a closed hand for 30–60 s showed slight anther opening and pollen release, which could be used for successful pollination. In addition, sterile anthers collected at 15 °C remained indehiscent in Eppendorf tubes for 30 min at room temperature; however, they subsequently opened and released viable pollen under the light/increased temperature from a dissecting microscope (Fig. 5F, G).

### Fertility restoration occurs before pollen mitosis stages

*HvMS1* overexpression plants grown at 15 °C were transferred at different floral developmental stages, based upon individual tiller staging (Fernández Gómez and Wilson, 2012), to controlled (20 °C; CE) and variable (>25°C; glasshouse) higher temperature conditions. *HvMS1OEx* plants transferred to higher temperatures with tillers at PMI or earlier [last flag elongation1 (LFE1) stage; Fernández Gómez and Wilson (2012)] recovered fertility. Fertility reached up to 100% in lines transferred during the early developmental stages, compared with those transferred after PMII (Fig. 4D), indicating that pollen developed beyond the PMI stage could not be rescued *in vivo* as pollen development had progressed too far. In the native WT situation, *HvMS1* is expressed transiently in the tapetum immediately prior to PMI; however, in the *HvMS1OEx* lines, the transgene is regulated by the Ubi1 promoter, resulting in increased, but also ectopic expression. The PMI stage is clearly important for the observed temperature rescue, indicating that the level of *HvMS1* at this stage is critical and impacts significantly on the subsequent development of the anther.

### The *HvMS1OEx* environmentally sensitive phenotype is conserved in other barley varieties

A single-copy Golden Promise *HvMS1* overexpression line was crossed with three different elite barley varieties, RAGT Planet, Moonshine, and Optic, to determine whether the *HvMS1*-induced sterility was consistently seen in different barley varieties. From backcross 1 to 4, the same sterile phenotype was observed in all varieties carrying the *HvMS1OEx* transgene at 15 °C. Fertility recovery was seen in overexpressing plants at >20 °C (Fig. 5I), as previously observed in the Golden Promise background.

## Discussion

### *HvMS1* overexpression is responsible for the male-sterile phenotype

We previously identified the orthologous gene of Arabidopsis *MS1* in barley (Fernández Gómez and Wilson, 2014), encoding a PHD-finger domain transcription factor that is essential for pollen formation and is expressed transiently in the tapetum immediately prior to PMI. Reducing expression of *HvMS1* by RNAi silencing in barley resulted in minor fertility reductions, whereas complete male sterility was observed by *HvMS1* overexpression (Fernández Gómez and Wilson, 2014). Here we have characterized this male sterility phenotype further and show that it is temperature dependent, which as far as we are aware is the first report of environmentally restorable male sterility in barley.

Complete male sterility was observed in *HvMS1OEx* lines, showing >60-fold increased *HvMS1* expression, compared with the native level (Fig. 1). Fertility in the *HvMS1OEx* lines could be rescued by reducing *HvMS1* expression to 15- to 30-fold above the native level, by a functional *HvMS1-RNAi* construct (Fig. 1), confirming that increased ectopic *HvMS1* expression is the principal cause of the observed male sterility.

Barley *HvMS1OEx* lines showed normal anther and pollen development until just prior to dehiscence; however, after this point, the anthers failed to open and were indehiscent (Fig. 1J, M; Supplementary Fig. S4). Viable pollen was present in these anthers (Fig. 5G); however, the pollen was extremely sticky. This suggests that *HvMS1* overexpression does not affect pollen development *per se*, but rather anther development and subsequent opening. The *HvMS1OEx* tapetum appeared active for longer and exhibited delayed degradation (Fig. 3), with associated increased deposition of materials onto the anther and pollen walls. The retention of the tapetum cell layer, alongside the increased pollen stickiness, may be preventing anther indehiscence and failure of pollen release. A similar phenotype was previously reported in *HvGAMYB* overexpression lines (Murray *et al.*, 2003), with failed anther dehiscence and sticky, albeit apparently viable, pollen. *OsGAMYB* has been shown to act upstream of *PTC1* (rice orthologue of *AtMS1*) (Fernández Gómez *et al.*, 2015) and therefore may be inducing expression of similar regulatory networks in the *HvMS1* overexpression lines. However, here we show that the male sterility linked to *HvMS1* overexpression is temperature sensitive, with fertility rescued by transfer to ≥20 °C before the PMI stage; as far as we are aware, temperature sensitivity in *GAMYB* overexpression lines has not been reported.

### Anther dehiscence and pollen release are temperature sensitive in *HvMS1* overexpression lines

Fertility recovery was observed in *HvMS1Ox* lines grown in, or transferred to, temperatures >20 °C (standard conditions 15 °C) (Fig. 2), provided this happened prior to the PMI stage of pollen development (Fig. 4). Restoration of fertility *in vivo* was developmentally specific, with lines transferred to higher temperatures after PMI remaining male sterile (Fig. 4D). Increased *HvMS1* expression was maintained under the higher temperature conditions (Fig. 2I, J) and in some cases higher levels of *MS1* expression were seen under the increased temperatures. However, very high *HvMS1* expression from homozygous lines, or lines with multiple copies of the *HvMS1* transgene, resulted in pleiotropic development and stunted plants (Supplementary Fig. S2), and in these cases fertility could not be rescued by increased temperature. It therefore appears that the increased temperature is not rescuing fertility by reducing *HvMS1* expression levels *per se*, as seen with the RNAi lines; rather it is mitigating the changes associated with increased induction of the downstream *MS1* regulatory network, or rescue of developmental progression, that are caused by ectopic high-level expression of *HvMS1*. Another possibility is that the MS1 protein may be particularly sensitive to higher temperatures and thus the HvMS1 protein could have reduced function at >20 °C, resulting in fertility rescue when the ectopic MS1 levels are reduced. However, qRT-PCR showed that *HvMS1* expression in A1.6, a sterile line homozygous for the *HvMS1OX* transgene grown at 22.5 °C (Fig. 2J), was greater than in equivalent single-copy sterile lines grown at 15 °C (Lines B1.3 and B1.5), and fertile lines grown at 22.5 °C (Lines A1.7, A1.11, and B1.8) (Fig. 2J). This suggests that HvMS1 is still functional at temperatures >20 °C, and can induce male sterility when the overexpression level is too high for fertility restoration with abnormal development.

Anther dehiscence is a highly regulated process that requires deposition of secondary thickening in the endothecium, enzymatic digestion of the septum and stomium, combined with differential endothecium and epidermal cell expansion and dehydration (Li *et al.*, 2011). Endothecium secondary thickening appears to form normally in the *HvMS1* overexpression lines regardless of the growth temperature (Fig. 3; Supplementary Figs S3, S4). However, enzymatic digestion of the septum and generation of single locules, which is essential prior to stomium breakage and dehiscence (Wilson *et al.*, 2011), does not occur normally in *HvMS1OEx* lines at 15 °C (Fig. 3B–D). In the event of total septum breakage (Supplementary Fig. 4a, red circle), the persistent tapetum appears to still hold the locules together and a single locule fails to form (Supplementary Fig. S4, red circle). Septum breakdown alone does not guarantee dehiscence and pollen release, since retraction of the anther walls, and other processes, such as pollen swelling and anther desiccation, are necessary for pollen release (Matsui *et al.*, 1999; Nelson *et al.*, 2012). Pollen swelling occurs in rice and barley just before dehiscence, which generates significant force to break the weakened septum (Matsui *et al.*, 2000). In *HvGAMYB* overexpression lines, the indehiscent anthers contain smaller and irregular pollen (Murray *et al.*, 2003) whereas, in the WT, pollen grains are more compact due to swelling occurring just before dehiscence (Matsui *et al*., 1999, 2000). Pollen in the *HvMS1* overexpression lines clumped together and were very sticky when manually excised from anthers. This implies an altered deposition of pollen wall materials and potential changes in anther locule dehydration. This, alongside the residual tapetum, could reduce the pressure exerted on the anther walls by the pollen and dehydration that is needed to drive anther opening (Nelson *et al.*, 2012).

The final phase of anther dehydration has an important role to facilitate dehiscence (Matsui *et al.*, 1999; Nelson *et al.*, 2012); this can be passive, via evaporation, or involve active removal of fluid from the anther (Keijzer, 1987; Pacini *et al.*, 2006). In *HvMS1* overexpression, changes in the anther wall structure, including delayed tapetum breakdown, or altered composition and increased deposition of pollen wall materials (Supplementary Fig. S4), may modify dehydration sufficiently to abort dehiscence at the lower (normal) temperature of 15 °C. Complete tapetum degeneration was observed at >20 °C (Fig. 3F–H) accompanied by normal anther dehiscence and fertility, which may reflect altered membrane fluidity, or increased dehydration, associated with the higher temperatures. In tomatoes, selective transformation of starch into sugars creates regions within the anther with increased osmotic potential, facilitating water removal (Bonner and Dickinson, 1989). In Arabidopsis, water has been shown to be removed from anthers through H^+^ ion sucrose transporters such as AtSUC1, which accumulate in the anther connective tissues (Stadler *et al.*, 1999). Aquaporins such as tobacco PIP1 and PIP2 have also been associated with cell to cell movement of water and dehydration and rehydration of the anther (Ruiter *et al.*, 1997; O’Brien *et al.*, 2002; Tyerman *et al.*, 2002; Soto *et al.*, 2008; Franchi *et al.*, 2011). Partial dehiscence could also be facilitated in late stage *HvMS1OEx* anthers by transient higher temperatures (microscope lights or enclosure in a hand); this may be a consequence of increased dehydration which generates sufficient energy for dehiscence despite the persistent tapetum and increased pollen wall deposits.

The *HvMS1* overexpression lines show an increased accumulation of materials in the locule, with altered anther wall formation and delayed tapetum breakdown. Tapetum breakdown appears to occur normally in the *HvMS1OEx* lines at higher temperatures (Fig. 3F–H). The increased temperature prior to PMI may be enhancing tapetum development and thus enabling timely tapetum breakdown and the cessation of pollen wall deposition, thus facilitating rescue of fertility at the higher temperatures. On the other hand, the late *in vitro* opening of anthers by temperature may provide additional force to overcome failure of dehiscence, regardless of pollen wall materials and tapetum retention, which are not achievable *in vivo*.

### *HvMS1* overexpression male fertility restoration is conserved in elite *Hordeum* varieties

The *HvMS1OEx*-induced male sterility and temperature-dependent rescue observed in barley variety Golden Promise was maintained when transferred into the different barley varieties tested (Fig. 5). Fertility recovery at 20 °C in Golden Promise (Fig. 4A–C) was consistently lower than the recovery observed for the other three varieties (Fig. 5I). This was attributed to the increased sensitivity of Golden Promise to high temperature; however, observed fertility differences were not major and no significant differences were observed in Golden Promise anther sections from plants grown at 15 °C and >22.5 °C (Fig. 3). Furthermore, RAGT Planet, Moonshine, and Optic showed advantages as potential female lines for hybrid breeding due to their floral morphology and anther extrusion. Golden Promise, as for the other three varieties, is self-pollinating; however, it fails to extrude its anthers. Cross-pollination also requires floral gaping to facilitate fertilization. *HvMS1* overexpression male-sterile lines (all four varieties) showed wide separation between lemma and palea, leaving the ovary exposed to cross-pollination as observed in Fig. 5A–E. This floral gaping was not a result of cleistogamy as no lodicule swelling was observed, but rather ovary swelling (Fig. 5A–C) which is initiated when the ovary has not been pollinated, (Nair *et al*, 2010), triggering ovary swelling and floral opening (Fig. 5D, E). Ovary swelling has been previously described in wheat as a response to self-pollination failure, in order to ensure seed set by cross-pollination (Okada *et al.*, 2018).

### Potential applications of HvMS1 overexpression for breeding

Hybrid seed production systems have been commercialized for a number of crops and have been valuable in contributing to increased production; however, most are species-specific approaches. Hybrid breeding success depends on the level of heterosis and the availability of a cost-effective hybrid seed production system (Ramage, 1983; Gowda *et al.*, 2010). For instance, it is very successful in cross-pollinated crops such as maize (*Zea mays* L.) (Duvick, 1999) because of high levels of heterosis and ease of emasculation, or rice (*Oryza sativa* L.) with a reliable CMS system and yield increases of 15–20% (Xiao *et al.*, 1995; Virmani and Kumar, 2004). To implement a hybrid breeding system, the problems of limiting self-fertilization and ensuring rescue of F_1_ and female line fertility need to be overcome. In addition, the male-sterile line (pollen receptor) needs to be maintained.

CHMS (chemical male sterility), CMS, or environmental-dependent hybrids have been widely used to prevent self-fertilization. CHMS has proved to be one of the most successful hybrid systems due to its simplicity and non-genetic basis, with most European hybrids generated using Croisor (Longin *et al.*, 2012). However, such systems are costly and dependent upon environmental conditions and genotypes (Parodi and Gaju, 2009), and regulatory constraints have prevented CHMS use globally (Longin *et al.*, 2012). CMS has been extensively used to increase rice production in China (Eckardt, 2006); *T. timopheevii* Zhuk.-derived male-sterile cytoplasm has also been used for commercial wheat hybrid production (Longin *et al.*, 2012). CMS has also been commercialized by Syngenta for barley hybrid breeding in Europe (Longin *et al.*, 2012). However, CMS is relatively inflexible, requiring a CMS line and an effective nuclear fertility restorer, and in some cases has been associated with yield penalties or other undesirable phenotypic or environmental effects (Kaul, 1988). This has been observed in *T. timopheevii* Zhuk.-derived male-sterile cytoplasm, which has shown incomplete fertility restoration, shrivelled F_1_ seed, and thus compromised yield (Adugna *et al.*, 2004). Nevertheless, the heterosis advantage has meant that this hybrid technology is being used for cultivation in an area of approximately 35 000 ha in India (Singh, 2010).

 Environmentally dependent hybrid systems rely on switchable male sterility, which consists of a maintainer line, that is sterile depending on photoperiod sensitivity (PGMS) or temperature sensitivity (TGMS) conditions, and a restorer line (Zhang *et al.*, 2013). These two-line hybrid systems simplify breeding and seed production, and are used in 10–20% of hybrid rice in China (Si *et al.*, 2011) and >50% of hybrid wheat (Longin *et al.*, 2012). Among the TGMS, PA64S, an *indica* variety, shows male sterility when grown at >23.5 °C, but is fertile between 21 °C and 23 °C. However, TGMS can be a problem due to the unpredictable environmental temperature, which can cause failure in hybrid seed production (Zhang *et al.*, 1994). PGMS benefits from stable photoperiod within a season, but is limited due to the lack of fertility-restoring germplasm and climatic conditions (Gonghai *et al.*, 2005). An example of a potential PGMS system is the *Carbon Starved Anther* locus, which regulates sugar partitioning (Gonghai *et al.*, 2005; Zhang *et al*., 2010, 2013). This mutant shows photoperiod sensitivity, with short day (SD) male sterility and long day (LD) fertility.

As far as we are aware, our *HvMS1* overexpression thermosensitive sterility is the first report of environmentally sensitive male sterility in barley, which therefore offers potential for reversible breeding systems in this important crop. For example, the heterozygous *HvMS1OEx* grown at 15 °C could be used as the female parent, enabling outcrossing with a selected male line. If the transgene is combined with a seed selectable marker, selection of the segregating non-transgenic seed could be ensured, enabling generation of hybrid, non-transgenic seed. The *HvMS1OEx* line could be maintained by growing at >20 °C to rescue fertility. In addition, *HvMS1* expression could be modified using alternative promoters to optimize the sterile thermosensitive phenotype, for instance making the homozygous double copy transgenic line restorable. In addition to hybrid seed production, the *HvMS1OEx* sterile line is a useful tool for crossing in breeding and research programmes, avoiding need for emasculation.

This work has demonstrated the impact of environmental conditions on the regulation of pollen development and release, and the application of transgenic approaches for the control of fertility. Understanding anther and pollen development is essential to control fertility for hybrid seed production. *HvMS1* is vital for pollen formation and its overexpression disrupts release of functional pollen as a consequence of prolonged tapetum activity, increased pollen wall material deposition, and failure of anther stomium and septum lysis at 15 °C. This indicates the importance of this gene and its associated network, and the impact of variable environmental conditions on pollen development.

## Supplementary data

The following supplementary data are available at *JXB* online.

Fig. S1. Genotyping data for transgenic lines.

Fig. S2. *HvMS1OEx* plants exhibited reduced height.

Fig. S3. Transverse sections of *HvMS1*-overexpressing and WT anthers.

Fig. S4. Transverse sections during anthesis of anthers from *HvMS1OEx* and wild-type plants grown at 15 °C.

Table S1. Primers used in this study.

eraa382_suppl_Supplementary_File001

## Data Availability

All data supporting the findings of this study are available within the paper and within its supplementary data published online.
