# Supplementary material for: Increased expression of the MALE STERILITY1 transcription factor gene results in temperature-sensitive male sterility in barley
Source: J Exp Bot. 2020 Aug 21;71(20):6328–39. doi: 10.1093/jxb/eraa382 (PMC7586743; doi:10.1093/jxb/eraa382)
Supplement: eraa382_suppl_Supplementary_File001 [file eraa382_suppl_supplementary_file001.pdf]

a

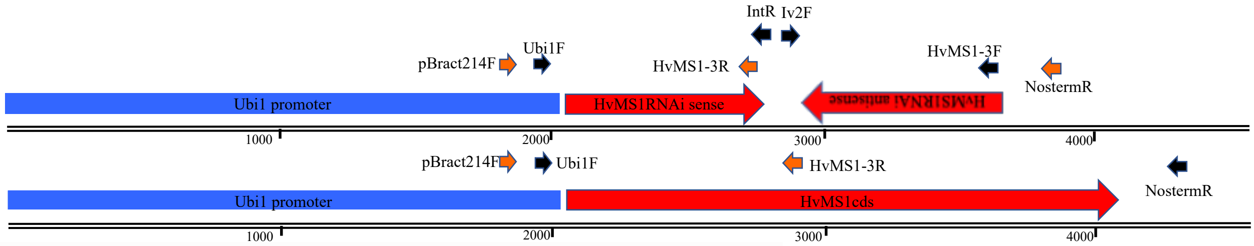

b

c

d

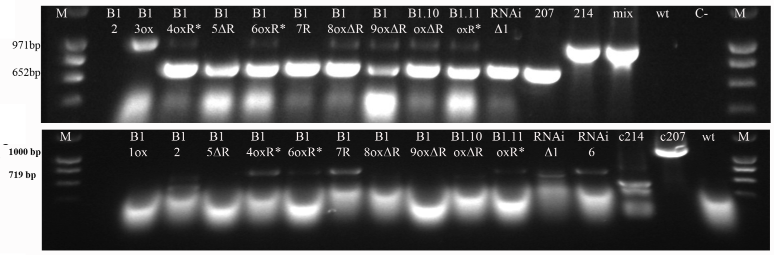

e

Transgene	Band Size (bp)	B1.1 ox	B1.2	B1.3 ox	B1.5 ΔR	B1.4 oxR*	B1.6 oxR*	B1.7 R	B1.8 oxΔR	B1.9 oxΔR	B1.10 oxΔR	B1.11 oxR*	RNAi. 1Δ	RNAi. 6
OEx	971	-	X	✔	X	✔	✔	X	✔	✔	✔	✔	X	X
RNAi-S	652	X	X	X	✔	✔	✔	✔	✔	✔	✔	✔	✔	✔
RNAi-AS	719	X	X	-	X	✔	✔	✔	X	X	X	✔	X	✔

f

**Line OEx x wt**	**OEx transgene**	**Phenotype**
B1.2	N	Fertile
B1.3ox	Y	Sterile

Line OEx x RNAi	OEx transgene	RNAi transgene Sense	RNAi transgene Antisense	Phenotype
B1.4 oxR*	Y	Y	Y	Fertile
B1.5 ΔR	N	Y	N	Fertile
B1.6 oxR*	Y	Y	Y	Fertile
B1.7 R	N	Y	Y	Fertile
B1.8 ΔR	Y	Y	N	Sterile
B1.9 oxΔR	Y	Y	N	Sterile
B1.10 oxΔR	Y	Y	N	Sterile
B1.11 oxR*	Y	Y	Y	Fertile

Supplementary Figure 1. Genotyping data for transgenic lines

a) pBract207::HvMS1RNAi construct. Small arrows indicate primers used to confirm presence of the sense and antisense silencing fragments (Ubi1F & InR, amplified 791 bp and Iv2F & NostermR amplified 719 bp).

b) pBract214::HvMS1 protein coding sequence OEX construct. pBract214F & HvMS1-3R primers were used to confirm the presence of the RNAi and the over-expression transgenes (652 and 971 bp, respectively).

c) PCR analysis of *HvMS1OEx* transgene and *HvMS1RNAi*-Sense inserts in the F1 segregating population from heterozygous *HvMS1OEx* sterile line crossed with wt (B1.2 and B1.3) and *HvMS1RNAi* (B1.4 to B1.11) lines. The *HvMS1OEx* transgene generated a 971 bp product (pBract214F and HvMS1-3R primers (Lines B1.3, 1.4, 1.6, 1.8-11)), no amplification was seen from wt, or after loss of the construct (e.g. Lines B1.2, 1.5, 1.7). Plants resulting from the double transgene crossing (OEx x RNAi) were also analysed for the RNAi sense fragment. Lines B1.4 to B1.11 contained the sense RNAi construct (pBract214F & HvMS1-3R, 652 bp product). Lines containing both the OEx and RNAi transgenes gave two bands (971 and 652 bp; Lines B1.4, B1.6, B1.8-11); 207: Positive Sense RNAi Control-pBract207::HvMS1RNAi; 214: Positive HvMS1OEx Control-pBract214::HvMS1cds; mix: mixed samples of 207 and 214; C-: water negative control; M: 1Kb ladder

d) PCR analysis of *HvMS1RNAi*-Antisense inserts in F1 segregating population from (c). Lines B1.4, B1.6, B1.7 and B1.11 contained both the Sense and Antisense RNAi inserts, whilst Lines B1.5, and B1.8-10, amplified only the sense insert (c) with the antisense missing (d). Lines RNAi: *HvMS1RNAi* genomic DNA control; RNAi.1 had only the sense fragment (c), whereas RNAi.6 has the antisense (d). c214 and c207: Empty destination vectors, pBract207 and pBract214; 207 showed a product of around 1100 bp, using primers iv2F and NostermF of the RNAi insertion site. No product was expected for pBract214.; wt: Barley gDNA.

e) PCR transgene genotyping; - not tested; √: Positive PCR for the transgene; x: Negative PCR for the transgene. Δ: deleted non-functional RNAi transgene; *indicates OEx and functional RNAi transgenes. All over-expression lines were heterozygous for the *HvMS1* over-expression transgene.

f) Genotyping and phenotyping of progeny from crosses between HvMS1 over-expression (OEx) lines with wt and HvMS1-RNAi silencing lines. Lines in which both HvMS1OEx and functional HvMS1RNAi were present were fertile, lines with OEx construct, or OEx and deleted RNAi construct were sterile. Y: Positive PCR for the transgene; X: Negative PCR for the transgene.

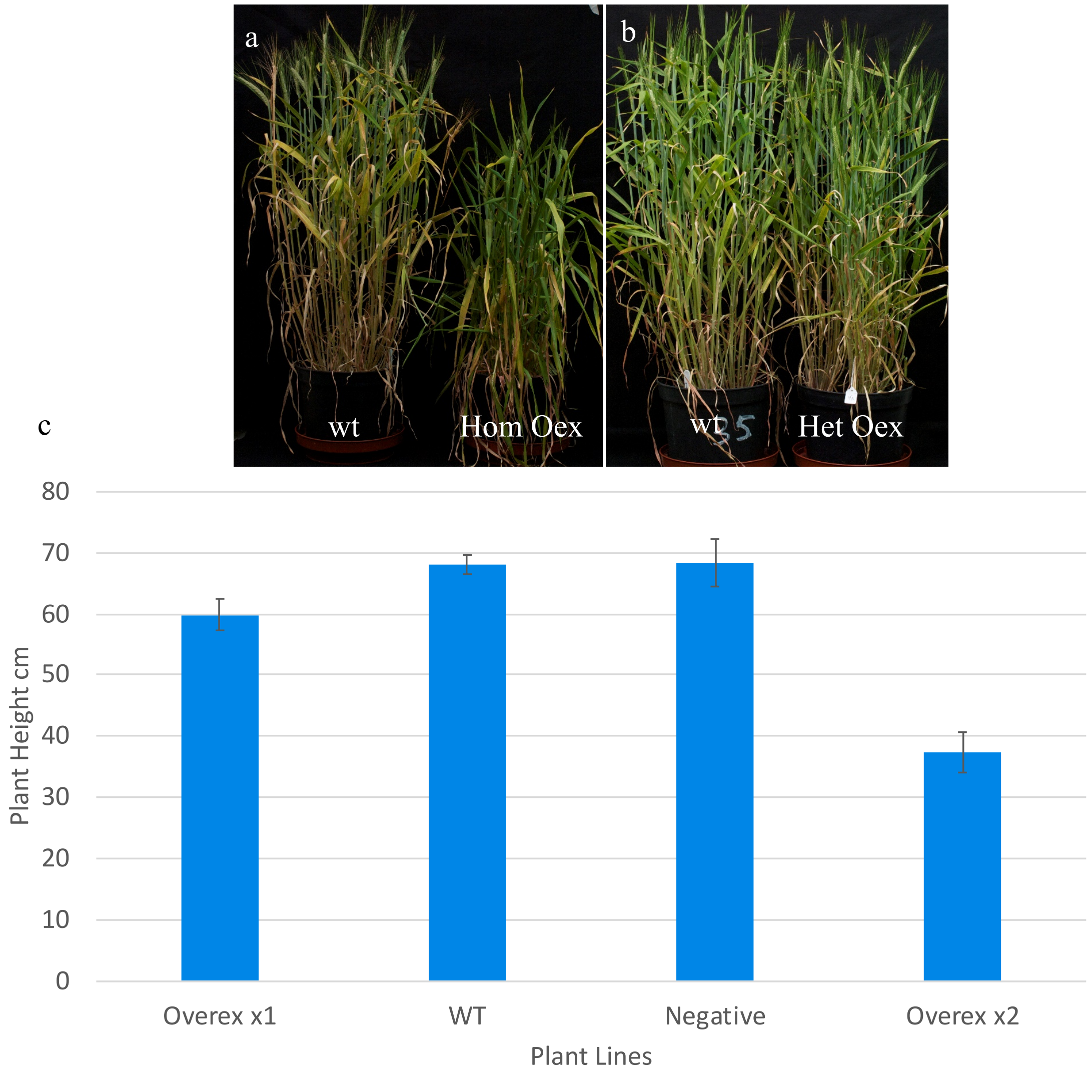

Supplementary Fig. 2 *HvMS1OEx* plants exhibited reduced height.

a) Comparison between wt and a homozygous *HvMS1OEx* line (two copies of *HvMS1OEx* (Hom OEx)). The Hom line shows major height reduction. b) Comparison between wt and a heterozygous *HvMS1OEx* line (one copy of *HvMS1OEx* (Het OEx)). The Het line is slightly smaller than wt. c) Plant height comparison between Het *HvMS1OEx*, wt, negative with no transgene after segregation and Hom for *HvMS1OEx*.

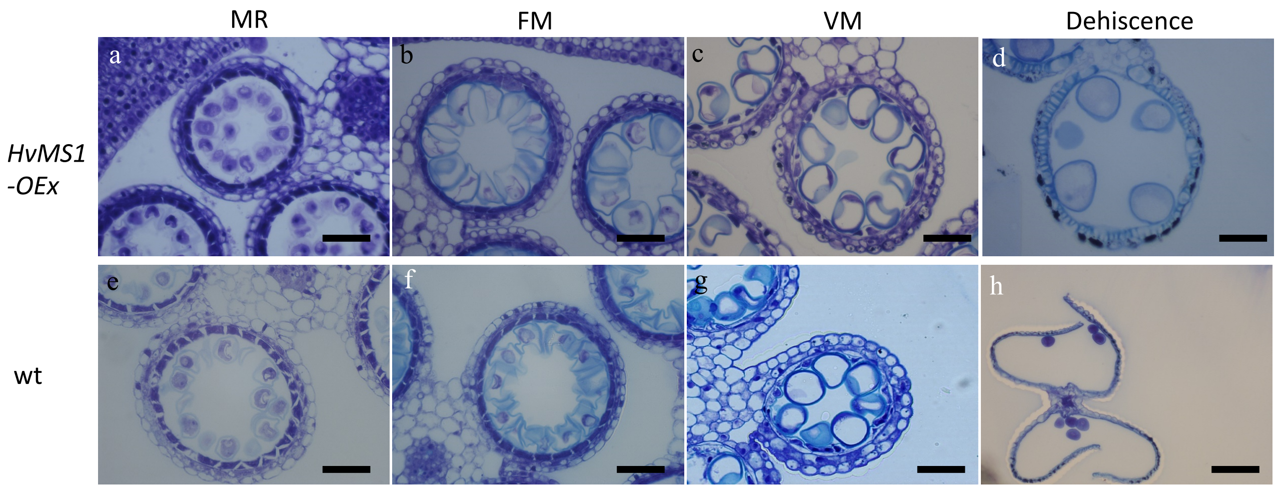

Supplementary Fig. 3. Transverse sections of *HvMS1* over-expression and wt anthers.

Anther and pollen formation follow a similar pattern in both over-expression (a-d) and wt (e-h). Failure of dehiscence was observed at the final stage of anther development in the *HvMS1OEx* lines (d) additional pollen wall material deposits and incomplete breakdown of the tapetum were seen in the *HvMS1OEx* anthers compared to wt (h). Anther developmental stages: MR: Microspore Release; FM: Free Microspores; VM: Vacuolated Microspores;. Scale bars: a-f:0.05mm; g: 0.1 mm; h: 0.2 mm.

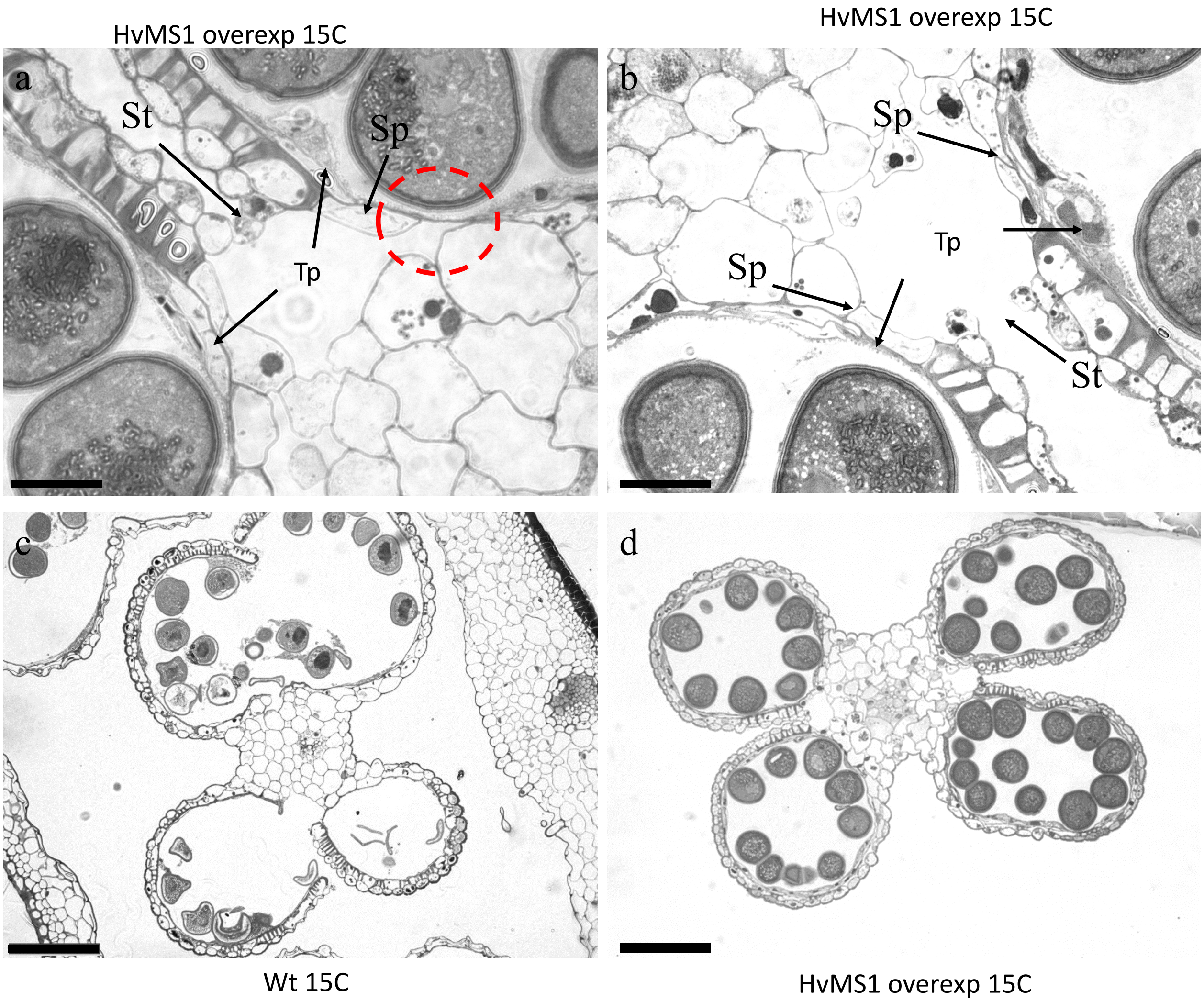

Supplementary Fig. 4 Transverse sections of anthers during anthesis from *HvMS1OEx* and wt plants grown at 15°C.

a-b) *HvMS1OEx* anthers fail to dehisce, septum degeneration is incomplete (b), however, regardless of septum breakdown ((a) red circle), the tapetum does not fully degrade which prevents anther locule opening. The stomium normally remains intact (a), however, complete rupture may occur (b), however this is not sufficient for anther dehiscence in the *HvMS1OEx* lines. Scale bar: 20µm c-d) Comparison between (c) wt and (d) *HvMS1OEx* (d) at 15°C. At this temperature, wt showed normal anther dehiscence (c) when compared with the indehiscence over-expression line (d). Scale bar: 100µm

Supplementary Table 1. Primers used in the study.

Primer	Primer Sequence
HvMS1-3R	CTGATGGCCTGGTACTTGGT
pBract214F	TTTAGCCCTGCCTTCATACG
Ubi1promF	ATGCTCACCCTGTTGTTTGG
i18R	CATCGTTGTATGCCACTGGA
ivF	CCAAAATTTGTTGATGTGCAG
NostermR	TGTTTGAACGATCCTGCTTG
HvMS1-1F	AGACCAAGTGCTGGTCGTTC
HvTubF	AGTGTCCTGTCCACCCACTC
HvTubR	AGCATGAAGTGGATCCTTGG
HygF	GCGAGAGCCTGACCTATTGC
HygR	CGGTGTCGTCCATCACAGTT
